# Automated Phenotypic Trait Extraction for Rice Plant Using Terrestrial Laser Scanning Data

**DOI:** 10.3390/s24134322

**Published:** 2024-07-03

**Authors:** Kexiao Wang, Xiaojun Pu, Bo Li

**Affiliations:** Institute of Agricultural Science and Technology Information, Chongqing Academy of Agricultural Sciences, Chongqing 401329, China; puxiaojunanna@163.com (X.P.); hnjzbobo@163.com (B.L.)

**Keywords:** phenotypic parameters, rice plant, automatic extraction, 3D point cloud, terrestrial laser scanning (TLS)

## Abstract

To quickly obtain rice plant phenotypic traits, this study put forward the computational process of six rice phenotype features (e.g., crown diameter, perimeter of stem, plant height, surface area, volume, and projected leaf area) using terrestrial laser scanning (TLS) data, and proposed the extraction method for the tiller number of rice plants. Specifically, for the first time, we designed and developed an automated phenotype extraction tool for rice plants with a three-layer architecture based on the PyQt5 framework and Open3D library. The results show that the linear coefficients of determination (R^2^) between the measured values and the extracted values marked a better reliability among the selected four verification features. The root mean square error (RMSE) of crown diameter, perimeter of stem, and plant height is stable at the centimeter level, and that of the tiller number is as low as 1.63. The relative root mean squared error (RRMSE) of crown diameter, plant height, and tiller number stays within 10%, and that of perimeter of stem is 18.29%. In addition, the user-friendly automatic extraction tool can efficiently extract the phenotypic features of rice plant, and provide a convenient tool for quickly gaining phenotypic trait features of rice plant point clouds. However, the comparison and verification of phenotype feature extraction results supported by more rice plant sample data, as well as the improvement of accuracy algorithms, remain as the focus of our future research. The study can offer a reference for crop phenotype extraction using 3D point clouds.

## 1. Introduction

Plant phenotypes, with genetic and environmental factors, are commonly used by plant breeders to meet specific breeding goals [1]. Quantitative evaluation of crop phenotype characteristics is an important aspect of crop breeding research, and is crucial for revealing the mechanism of biological traits [2,3]. The bottleneck of plant phenotype research lies in determining how to quickly obtain enough plant phenotype features [4]. Traditional methods for extracting crop plant phenotypic features often need manual measurement, which are time-consuming and labor intensive [5]. In recent years, traditional radiation transmittance measurements have been widely applied in related research, but the sensors could only obtain two-dimensional (2D) images of the target crop, thus gaining limited phenotypic information [6].

The emergence of light detection and ranging (LiDAR) technology, which can generate the accurate three-dimensional (3D) information of the target, has provided technical support for obtaining the structural information of scanning targets at a higher level of detail [7]. Currently, its scope has spread across a wide range of research areas, especially in agriculture [8]. As one of the agricultural application fields of LiDAR, crop phenotype feature extraction had also become a research focus of many scholars. Among the research on crop population phenotypes, many studies concentrated on crop canopy height [9,10,11], canopy biomass [12,13,14], and leaf area index (LAI) [15,16] using unmanned aerial vehicle (UAV) systems. Luo et al. estimated maize LAI, canopy height, and aboveground biomass using the combined hyperspectral imagery and LiDAR pseudo-waveforms, showing the strong liner correlation between LiDAR variables and LAI, height, and biomass [17]. Zhou et al. obtained the height of the maize canopy using a canopy height model based on the UAV-LiDAR data at different phases with higher estimation accuracy [18]. Compared with airborne LiDAR, terrestrial laser scanning (TLS) could provide precise, high-density, and repeatable data acquisition for monitoring specific crops with a cost-effective and easy to perform approach [19], which has been widely used in the precise extraction of individual crop phenotypic features. Shi et al. automatically measured the corn plant location and spacing by TLS, with a total plant counting error of 5.5% and a RMSE in spacing measurement of 1.9 cm [20]. Guo et al. estimated the wheat height using TLS, pointing out the 95th height percentile, H_95_, can effectively monitor the height during the entire growth stages, and at which the wheat height can be accurately detected for heights as low as 0.18 m [21]. Jin et al. proposed a median normalized-vector growth (MNVG) algorithm, which can segment the stem and leaf of the maize samples with different heights, degrees of compactness, leaf numbers, and densities from three growing stages using terrestrial LiDAR data [22]. Su et al. calculated plant height, plant area index (PAI), and projected leaf area (PLA) from the point clouds of different maize varieties under drought stress collected by TLS at the individual plant level [23]. There are many related studies of this kind, which have shown great potentiality in high-precision acquisition of crop features. However, relevant research has mostly focused on the population characteristics at the canopy level of crops, as well as the extraction of individual phenotypic features of plants such as maize, rapeseed [24], wheat [25], cabbage [26], and soybean [27]. As one of the important food crops, rice plays an important role in preserving food security and social stability. The rice plant has flat leaves, numerous tiller branches, and complex canopy structures, which pose significant difficulties in extracting its phenotypic features. Therefore, accurate extraction of rice plant phenotypes is of great significance in monitoring rice growth and crop breeding.

In this study, we put forward the automatic computational process of seven rice phenotype features by introducing multi-step point cloud processing algorithms, developed a plant phenotype feature extraction tool based on high-precision TLS data, and achieved the automatic extraction of rice plant parameters. This study can provide a reference for the analysis of rice crop characteristics. The main contributions of this study are as follows:

(1) Giving an automatic calculation acquisition approach to obtain the phenotype features of rice plants from a rice plant point cloud, including crown diameter, perimeter of stem, plant height, surface area, plant volume and PLA, and tiller number. We also proposed a point cloud extraction method for the tiller number for rice plants.
(2) Combined with PyQt5 and the Open3D library for processing 3D point cloud data, and integrating the related point cloud data algorithms, we purposefully designed the first tool for automatically obtaining phenotype parameters of rice plants, which is conducive to promoting the application of 3D point cloud technology in rice plant breeding.

## 2. Indicator Determination

Plant height, PLA, and plant volume are important metrics to estimate crop yield [26]. With the growth of the plant stem and increase in the tiller number, the plant canopy and leaf area accumulate a large amount of organic matter for the later nutritional and reproductive growth of rice. The plant surface area reflects the photosynthetic capacity of crop plants. Meanwhile, the crown diameter and the perimeter of the stem are closely related to plant biomass and plant growth status to some extent [28]. The tiller number of rice is an important factor reflecting the yield potential [29]. Therefore, combining the characteristics of the rice plant, this study selected seven phenotype features closely related to the rice plant biomass, photosynthesis, and yield as the phenotype target features of the rice plant to carry out the tool design.

**Figure 1:**
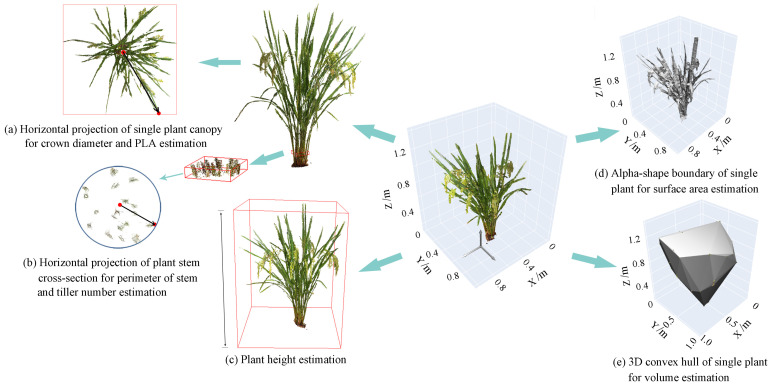
Schematic diagram of rice plant phenotypic feature.

The feature indicators of the rice plant are shown in Figure 1. The crown diameter is the maximum radius of the plant canopy from the horizontal centroid, and PLA is the percentage of the horizontal projected area in the minimum bounding rectangle of the canopy (Figure 1a). In Figure 1b, the perimeter of stem and tiller number are obtained by calculating the perimeter and number of branches at the section of the plant stem. The plant height is the vertical distance from the highest point of the plant to the reference point (Figure 1c). The surface area and volume are the surface area of the 3D model of the plant body (Figure 1d) and the volume enclosed by the minimum convex hull (Figure 1e), respectively. Table 1 presents the specific indicator algorithm outline used in this study.

## 3. Methodology

### 3.1. Overview

**Figure 2:**
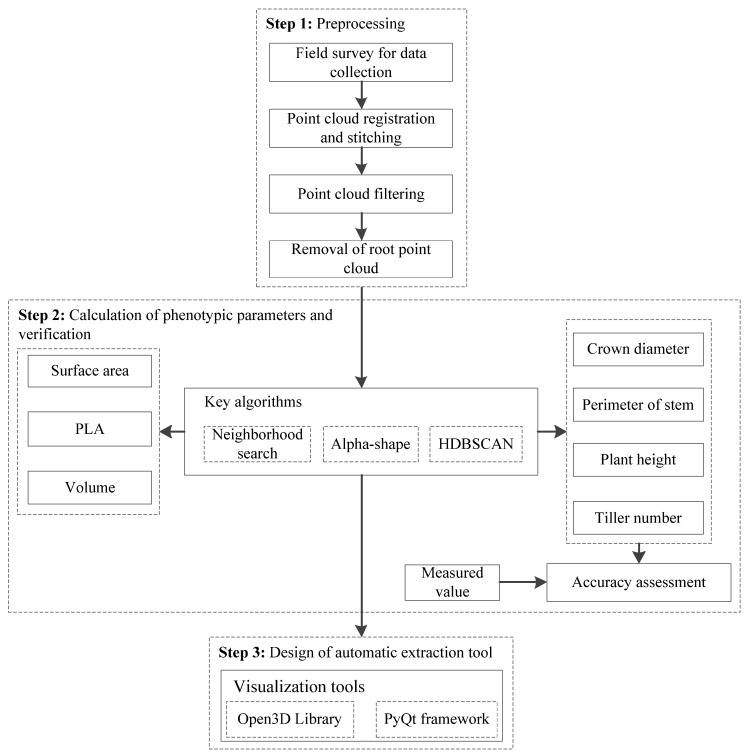
Flow chart of the study.

The flow chart of this study is presented in Figure 2. After the point cloud data collection and preprocessing, key algorithms of point cloud neighborhood search, Alpha-shape, and Hierarchical Density-Based Spatial Clustering of Applications with Noise (HDBSCAN) were utilized to extract the relevant phenotype parameters. Meanwhile, the reliability validation was conveniently conducted on the extraction results of the four indicators for field measurement. Finally, a visual tool for automatic extraction of rice phenotypic parameters was designed based on the Open3D library and PyQt framework.

### 3.2. Data Acquisition and Processing

**Figure 3:**
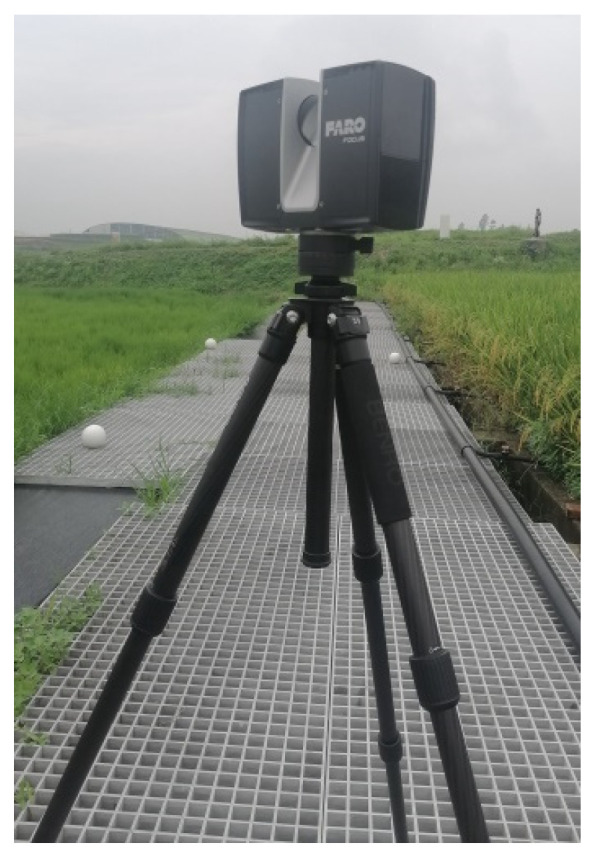
TLS sensor for data collection.

The point cloud data of rice plants were obtained indoors by the TLS equipment called FARO Focus S70, with an angle resolution of 0.009° and measurement error between 0.1 and 1.3 mm (Figure 3). To obtain the relatively complete plant point cloud data, the scanner was set up on both sides 1.5 m from the crop plants, with fixed scanning resolution of 1/4, quality level of 3× and scanning size of 10240 × 4267 Pt. Three target balls with a diameter of 15 cm were used for point cloud data registration. The registration process was completed in Faro Scene software 2020 with the fitting standard deviation of the balls within 1.5 mm. Then, objects unrelated to the plant were manually removed, and statistical outlier removal (SOR) filtering was used to clean the noise of the point cloud. Lastly, after removing the root point cloud, the “PCD cloud” format was exported for reading using Open3D library.

In addition, the crown diameter, perimeter of stem, and plant height of rice plants were obtained using a measuring tape, and the tiller number was obtained through manual counting. Assuming a measurement error of 1 cm, the average of three measured values was selected as the final value. The crown diameter was gained by measuring the diameter in both the maximum and minimum crown directions, calculating the average value, and converting it into a radius. The circumference of the stem was measured 3 cm above the root to obtain the perimeter of stem. Plant height was measured as the vertical distance from the root stem junction to the top of the plant.

### 3.3. Key Algorithms

#### 3.3.1. Neighborhood Search

Kd-tree [30] is a binary search tree which is commonly used to establish high-dimensional spatial indexes in k-dimensional data space, to achieve the construction of geometric topology information between discrete point clouds and thereby find the nearest neighbors of query points. In this study, the neighborhood search algorithm was used to search for the distance to calculate the perimeter of the stem and crown diameter of rice plants. On the basis of constructing a point cloud Kd-tree, the geometric topological relationships between discrete points were established to conduct a point cloud neighborhood search. This study made use of the radius neighborhood search algorithm to traverse the centroid and all horizontal projection points with a step having a radius of 2 cm, and returned the search radius corresponding to its farthest point. The main flow of the radius neighborhood search module is shown in Algorithm 1.
**Algorithm 1.** Radius neighborhood search algorithm.**Input:** The point cloud of stem cross-section or rice plant1: Project the point data onto the X-Y plane2: Calculate the centroid position of the horizontal projection point: **Z(**$x_{i,}\;y_{i}$**)**3: Count the horizontal projection point cloud: ***n***
4: Build Kd-tree structure for point clouds5: Set initial search radius and step size6: **for** R in the radius range7:  Neighborhood search with Z($x_{i,}{\;y}_{i}$) as the center and R as the radius, and record the number of points searched: **k**8:  if **k** = ***n***:9:   return R10: **end****Output:** Calculate the perimeter of stem or crown diameter of rice plant

#### 3.3.2. Three-Dimensional Surface Reconstruction

**Figure 4:**
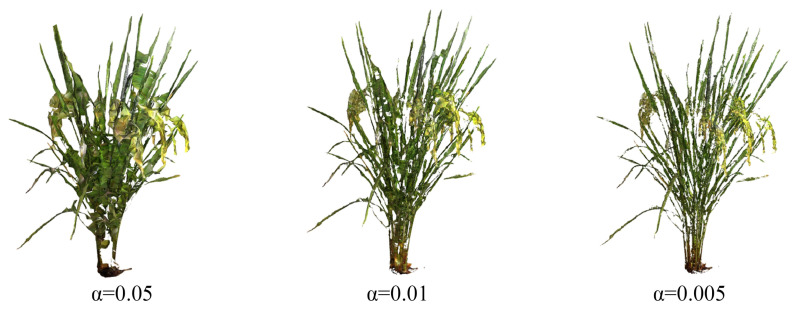
Three-dimensional reconstruction model of rice plants under different α values.

Alpha-shape [31] is a boundary point extraction algorithm proposed to construct a ball with the radius α based on three points in space. The ball is used to determine the boundary of the point cloud model. The basic Alpha-shape algorithm relies on the Delaunay triangulation, in which each triangle edge is characterized with a radius α of the smallest empty circle containing the edge or triangle [32]. Assuming that S is a finite point set in 3D space, and α is a real number (α∈[0, ∞)). Alpha-shape is the convex hull of S point set when α = ∞. Along with α value reduction, the ball gradually generates pits or holes, and the shape of the target tends to become more refined. This study mainly utilized the Alpha-shape algorithm to complete the 3D surface reconstruction process of rice plants and further extract the plant surface area. By comparison, the range of α was set from 0 to 2, with an initial value of 0.0040 and increasing step size of 0.0005. The 3D surface reconstruction effect of the rice plant corresponding to the value of 0.05, 0.01, and 0.005 is shown in Figure 4.

#### 3.3.3. Point Cloud Density Clustering

The HDBSCAN algorithm is a data clustering method proposed by Campello [33], which combines both hierarchical clustering and density segmentation. It extends the Density-Based Spatial Clustering of Application with Noise (DBSCAN) algorithm by converting it into a hierarchical clustering algorithm to extract a flat clustering based on the stability of clusters, which is more robust to parameter selection. The basic principle is to construct a reachable graph by calculating the reachable distance between adjacent points and the core point, and finally introduce hierarchical clustering and cluster tree compression to obtain the final cluster. The reachable distance between two points is shown in Formula (1).

(1) $$d_{k}\left(p,q\right)=max\left\{c_{k}\left(p\right),c_{k}\left(q\right),d\left(p,q\right)\right\}$$

where $d\left(p,q\right)\;$is the original metric distance between *p* and *q*, and the core distance $c_{k}\left(p\right)=d\left(p,N^{k}\left(p\right)\right)$ represents the distance between the core point *p* and the *k*-th neighboring point.

Because of the tiller branches in the stem of the rice plant and the high-density point clouds, this study introduced the HDBSCAN algorithm to cluster the point clouds of the plant stem to obtain the tiller number. The algorithm was mainly implemented by calling the Python library “hdbscan” for point cloud data clustering programming. The implementation process can be decomposed into five steps [34]: (1) transform the space according to the density, (2) build the minimum spanning tree of the distance weighted graph, (3) construct a cluster hierarchy of connected components, (4) condense the cluster hierarchy based on the minimum cluster size, and (5) extract the stable clusters.

### 3.4. Accuracy Evaluation

In this study, a linear function was adopted to fit the relationships between the extracted values and manually measured values, and the statistical indicators *R*^2^, *RMSE*, and *RRMSE* were utilized to assess the relationships. The model performance was more accurate with *R*^2^ near to 1, and *RMSE* and *RRMSE* near to 0. *RRMSE* represents the degree of difference between the extracted and the measured values (*RRMSE* < 10% indicates no difference, 10% ≤ *RRMSE* < 20% denotes a small difference, 20% ≤ *RRMSE* < 30% is moderate, and *RRMSE* ≥ 30% represents a large difference [35]. The calculation formulas of *R*^2^, *RMSE*, and *RRMSE* are shown in Formulas (2)–(4):

(2) $$R^{2}=1-\frac{\sum_{i=1}^{n}{\left({\hat{x}}_{i}-\bar{x}\right)}^{2}}{\sum_{i=1}^{n}{\left(x_{i}-\bar{x}\right)}^{2}}$$

(3) $$RMSE=\sqrt{\frac{\sum_{i=1}^{n}{\left({\hat{x}}_{i}-x_{i}\right)}^{2}}{n}}$$

(4) $$RRMSE=\frac{RMSE}{\bar{x}}\times 100\%$$

where $x_{i}$ and $\bar{x}$ represent the measured value and the average of the measured values, respectively, ${\hat{x}}_{i}$ represents the extracted value, and n represents the number of samples.

### 3.5. Visualization Tools

#### 3.5.1. Open3D Library

Open3D is a high-performance open-source library for fast processing of 3D point clouds developed by Intel Labs [36], supporting Python and C++ development environments. The main core functions of Open3D library include 3D data structures, 3D data processing algorithms, 3D scene reconstruction, 3D visualization, and 3D machine learning. The extraction of rice plant phenotype parameters in this visualization tool mainly involved its input/output module, geometric processing module, and visualization module.

#### 3.5.2. PyQt Framework

Qt is a cross-platform C++ Graphical User Interface (GUI) application development framework, which integrates numerous form controls. PyQt is the Python interface of the graphical programming framework, which can call the Application Programming Interface (API) function for the GUI system design and development through Python. With the connection mechanism between signals and slots, PyQt5 integrates multiple classes, which are distributed across multiple functional modules. In this study, the design of GUI was completed by the Qt Designer tool, which mainly involved the object classes such as “QMainWindow” class, “QAlication class”, “QDialog” class, and “QWidget” class. The modules mainly included “QtCore”, “QtGui”, “QtWidgets”, “QFileDialog”, and “QInputDialog”. The form controls mainly included the “QMenuBar” control for displaying functional menus, the “QLabel” control for indicator labels, and the “QTextBrowser” control for extraction results.

## 4. Results

### 4.1. Extraction Result Validation

**Figure 5:**
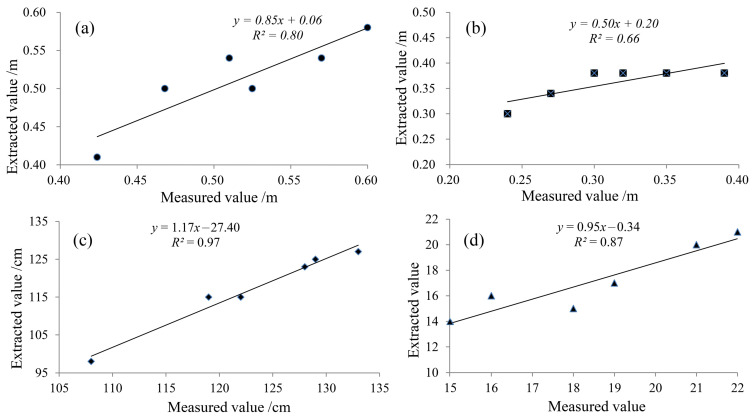
Scatter plots of the extracted and manually measured values of the rice plant. (**a**) Crown diameter; (**b**) perimeter of stem; (**c**) plant height and (**d**) tiller number.

Due to the need for professional instruments to measure the indicators such as plant surface area, PLA, and plant volume, this study conveniently selected the four indicators for field measurement to verify the extraction accuracy. The measured values were employed as standard values to compare with the extracted results of the rice plant phenotype parameters. As shown in Figure 5, the *R*^2^ values of the linear correlation between the measured value and the extracted values of crown diameter and tiller number are all above 0.80, and that of plant height is 0.97, which indicates the stronger correlation. The *R*^2^ of perimeter of stem is 0.66, slightly lower than other indicators, which may be caused by the difference between selecting stem slices and manually measuring stem position. Table 2 gives the *RMSE* and *RRMSE* of the four indicators. The *RMSE* of crown diameter, perimeter of stem, and plant height are maintained at the level of centimeter, and the tiller number is only 1.63. The *RRMSE* of crown diameter, plant height, and tiller number stay within 10%, indicating no obvious difference. However, the value of perimeter of stem is 18.29%, a small difference from the actual observations, owing to the possible differences in measurement positions. On the whole, the extraction algorithm embedded in the tool exhibits better robustness and can accurately extract the corresponding phenotypic characteristic parameters of rice plants.

### 4.2. Implementation of Automatic Extraction Tool

#### 4.2.1. Overall Structural Design

**Figure 6:**
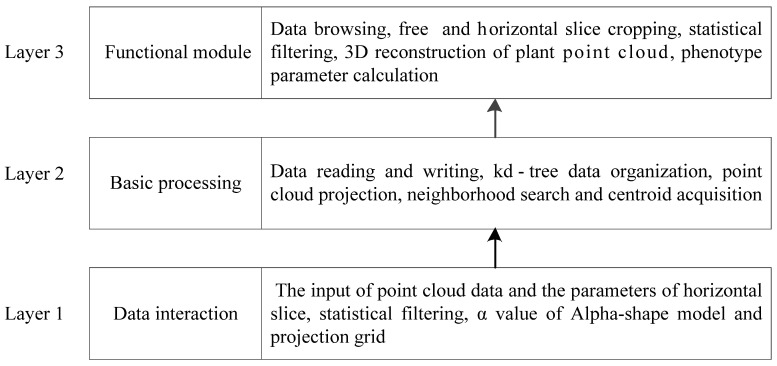
Architecture of rice plant phenotypic parameter extraction system.

For automatic extraction of rice plant features and display, this study employed the technologies of fast processing of 3D point cloud data and computer visualization programming to build the plant parameter extraction tool. Following the principles of a simple interface and convenient operation, the architecture of the rice plant phenotype feature automatic extraction tool was divided into three parts: data interaction layer, basic processing layer, and functional module layer. The overall architecture is shown in Figure 6.

- The data interaction layer mainly enables necessary data entry. The input data are mainly the plant point cloud datasets and some function parameters, such as the slice height of the point cloud, α value of the Alpha-shape algorithm, and the grid size of point cloud projection.
- The basic processing layer mainly involves the processing of input point cloud data, including reading and writing, organization, plane projection transformation, and centroid acquisition of point cloud data. These processes mainly provide basic data for implementing the subsequent main functional modules.
- The functional module layer is the key of the automatic extraction of rice plant phenotype parameters. The functions include data visualization browsing, free cropping and horizontal slice cropping, statistical filtering, surface model reconstruction, and automatic calculation and display of plant phenotype parameters.

#### 4.2.2. Division of Functional Module

**Figure 7:**
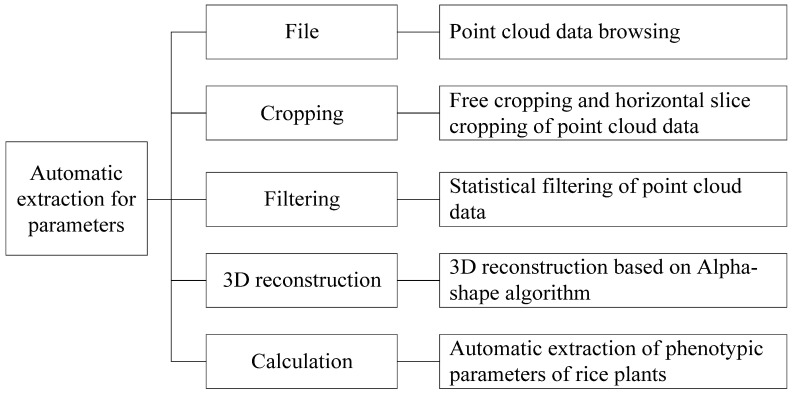
System menu function module.

Based on the architecture, the rice plant phenotype parameter extraction system can be divided into five menu functional modules: point cloud data browsing, data cropping, point cloud filtering, plant 3D reconstruction, and phenotype parameter calculation. The detailed description of each module is shown in Figure 7. After executing the corresponding module according to the requirements, automatic extraction and display of rice plant phenotype parameters can be achieved by inputting a small number of parameters.

#### 4.2.3. Implementation of Automatic Extraction Function

The processing and visualization of point cloud data mainly relies on the Open3D library, machine learning scikit-learn library, and visualization graphics framework PyQt5. The development environment was the integrated development environment PyCharm 2023.2.1 under Win 10, paired with the open-source Python package manager “Anconda3”.

**Figure 8:**
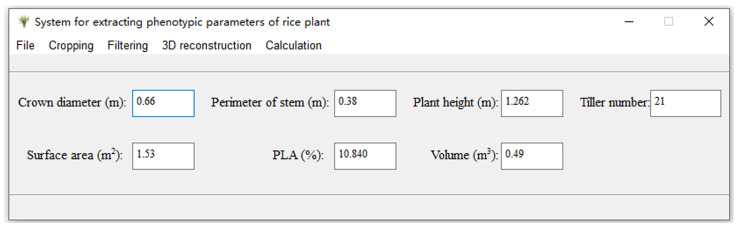
GUI of the extraction tool.

- The parameter calculation module was mainly used for the calculation and display of phenotypic parameter results. The slot function included the parameter calculation process, and the corresponding label objects and display text box objects were designed to label and display the calculation results. During the execution process of indicator calculation, the corresponding phenotype parameters were automatically calculated and displayed in view of the input of plant point cloud data, stem trimming point cloud data, α value, and point cloud projection grid spacing (Figure 8).

## 5. Discussion

For a long time, due to the complexity of crop plant morphology and structure, it has been generally difficult to obtain its surface area and volume. Meanwhile, field measurement of PLA also requires professional instruments and equipment. The technology of LiDAR can effectively reflect the vertical structural characteristics of crops, which is conducive to the construction of the plant morphology and the extraction of structure parameters [26]. In this study, we developed a rice plant phenotype parameter extraction tool, and achieved the fast and efficient acquisition of rice plant phenotype parameters, even some that are difficult to obtain using traditional methods. Moreover, we selected the parameters of crown diameter, perimeter of stem, plant height, and tiller number as validation indicators to verify the accuracy, with the result of good correlation and high reliability.

**Figure 9:**
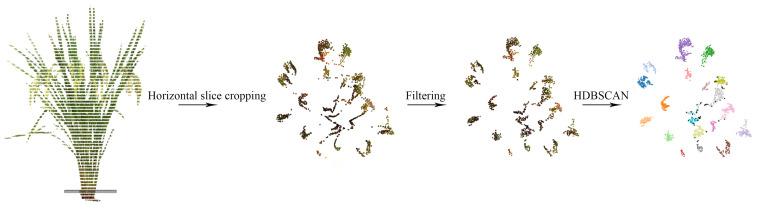
Extraction process of tiller number in rice plants. Different colors represent point cloud clusters on different tiller branches.

Nonetheless, the feature extraction results may be influenced by the following factors. Firstly, we designed the horizontal projection centroid of the stem section as the horizontal centroid of the crown for the point cloud neighborhood search to calculate the crown diameter. Therefore, it is necessary to ensure consistency between the two centers. It is thus important to ensure the plants are placed as vertically as possible when collecting the crop plant point cloud data. Secondly, the *RRMSE* between the extracted and measured values of stem circumference in this study is slightly larger than other indicators, because it is difficult to ensure the selected stem slices are consistent with the actual observed stem parts. Finally, generally speaking, a rice plant may have 15~20 tiller branches. Because of the high number of tillers in rice plants, the rice branches and trunks are severely obstructed, and the point clouds are incomplete. Considering the aggregation and the continuity in the horizontal and vertical direction, respectively, in the stem slice point clouds, this study used the HDBSCAN algorithm to perform density segmentation and counting after the process of statistical filtering (Figure 9). This study achieved the efficient extraction of tiller numbers from the rice plant, providing assistance for rice biological breeding.

In addition, this study only used the feature extraction results of six rice plant sample point clouds for accuracy verification, with a relatively small sample size, which is also a limitation of this study. Next, we will systematically collect more plant data to carry out feature extraction and comparison verification of corresponding phenotype parameters, and further improve the accuracy of rice plant phenotype parameter extraction by designing corresponding algorithms.

## 6. Conclusions

In this study, we identified seven phenotypic parameters of rice plants on the basis of their characteristics, and specially proposed the methods for obtaining the tiller number. Then, a rather complete rice plant phenotype parameter extraction tool with a three-layer framework for TLS point cloud data was built based on the PyQt5 visualization framework and the Open3D library. The visual tool can achieve the automatic extraction of rice plant parameters such as crown diameter, perimeter of stem, plant height, surface area, volume, PLA, and tiller number. The results show the R^2^ of crown diameter, tiller number, and plant height reached 0.80, 0.87, and 0.97, respectively, with that of perimeter of stem reaching 0.66. The *RMSE* of crown diameter, perimeter of stem and plant height was stable at the centimeter scale, and that of the tiller number was as low as 1.63. The *RRMSE* of crown diameter, plant height, and tiller number stayed within 10%, and the value of perimeter of stem was 18.29%, which indicated a high level of reliability. The tool developed in this study can achieve the goal of obtaining rice plant phenotype parameters quickly, accurately, and efficiently, which helps to improve the efficiency of rice breeding work. Although this tool is designed for rice plants, its application is not limited to rice. The extraction function of most indicators has a wide range of applicability. Additionally, the comparison and verification of phenotype feature extraction results supported by more rice plant sample data, as well as the improvement of accuracy algorithms, will be the focus of our next research.

## Figures and Tables

**Table 1:** Algorithm approach of phenotypic indicators for rice plants.

Indicators	Algorithm Outline
Crown diameter	Search for the horizontal distance from horizontal centroid of the stem cross-section to the farthest point of the projection of the canopy.
Perimeter of stem	Horizontally cut the plant stem, select the target stem section and project it onto the horizontal plane. Then use “neighborhood search algorithm” to search for the horizontal distance from its centroid to the farthest point, and regard it as the radius to calculate the circumference.
Plant height	Obtain the vertical distance from the highest point of the plant to the reference point.
Surface area	Convert the point cloud of plant to a surface model, and then calculate its surface area by the Alpha-shape boundary algorithm.
Volume	Calculate the volume of the minimum convex hull that surrounds crop plant.
PLA	Calculate the projection area of plant using the grid method, and the minimum bounding rectangle area by oriented bounding box (OBB) of the canopy horizontal projection.
Tiller number	Cut the stem horizontally into slices to get point cloud data of the target stem section, carry statistical filtering and hierarchical density clustering segmentation and count.

**Table 2:** *RMSE* and *RRMSE* of various indicators in the study.

Indicators	*RMSE*	*RRMSE*
Crown diameter	0.03 m	5.03%
Perimeter of stem	0.06 m	18.29%
Plant height	6.35 cm	5.16%
Tiller number	1.63	8.82%

## Data Availability

The scripts and datasets used and/or analyzed during the current study are available from the corresponding author on reasonable request.
